# Intraoperative Hemostatic Agents in Thoracic Aortic Surgery—A Scoping Review

**DOI:** 10.3390/jcm14114001

**Published:** 2025-06-05

**Authors:** Maite M. T. van Haeren, Caitlin Bozic, Jennifer S. Breel, Susanne Eberl, Faridi S. Jamaludin, Denise P. Veelo, Marcella C. A. Müller, Alexander P. J. Vlaar, Henning Hermanns

**Affiliations:** 1 Department of Anesthesiology, Amsterdam University Medical Centers, 1105 AZ Amsterdam, The Netherlands; 2 Department of Intensive Care, Amsterdam University Medical Centers, 1105 AZ Amsterdam, The Netherlands; m.c.muller@amsterdamumc.nl (M.C.A.M.);; 3 Medical Library, Amsterdam University Medical Centers, Location University of Amsterdam, 1105 AZ Amsterdam, The Netherlands; f.s.vanetten@amsterdamumc.nl

**Keywords:** coagulation, transfusion, hemostatic, thoracic aortic surgery, dissection

## Abstract

**Background/Objectives:** Patients undergoing open thoracic aortic surgery have the highest bleeding complication rates within cardiac–vascular surgery, but research on coagulation management mostly targets general cardiac surgery. This scoping review evaluates current evidence on intraoperative hemostatic agents and their effect on bleeding and blood transfusions in these patients. **Methods**: We searched MEDLINE (PubMed), Embase, and Cochrane Library on 2 July 2024. Eligible studies included randomized controlled (RCT) and observational trials with a comparison group and at least a sub-analysis regarding thoracic aortic surgery (excluding thoracoabdominal and isolated descending aorta surgery). **Results:** Our search yielded 4697 articles, with 33 included. These covered antifibrinolytics (3 RCTs, 10 observational studies), fibrinogen supplementation (3 RCTs, 4 observational studies), recombinant factor VIIa (rFVIIa, 8 observational studies), blood products (3 observational studies), and factor eight inhibitor bypassing activity (FEIBA, 1 RCT, 1 observational study). The impact of blood product transfusion on bleeding control is unclear due to a lack of placebo or no-transfusion comparisons, though it appears associated with more complications. Both FEIBA studies suggest reduced blood product use in aortic dissection surgery—one as rescue therapy, the other as standard treatment. Evidence on fibrinogen supplementation is mixed: a multicenter RCT showed increased transfusions, while smaller RCTs and observational studies showed reductions, possibly due to differences in pretreatment fibrinogen levels and patient selection. Observational studies on rFVIIa show conflicting results, likely due to selection bias. Two small RCTs—one on TXA, one on aprotinin—suggest reduced transfusions and blood loss. Comparative studies of different types of antifibrinolytics yielded conflicting results. **Conclusions**: Evidence on hemostatic agents in thoracic aortic surgery is limited. Small studies suggest potential for the routine use of antifibrinolytics, FEIBA, and fibrinogen supplementation—but only in bleeding patients with hypofibrinogenemia. High-quality RCTs focused on thoracic aortic procedures are needed to determine optimal coagulation management.

## 1. Introduction

Patients undergoing open thoracic aortic surgery often have major bleeding complications requiring appropriate coagulation management [1]. Thoracic aortic surgery requires the use of cardiopulmonary bypass (CPB), where the foreign materials can activate the coagulation cascade. Heparin as an anticoagulant is, thus, routinely used and antagonized with protamine after CPB to restore hemostasis [2,3,4]. Nevertheless, both coagulopathy and massive bleeding can occur after CPB [5,6]. These bleeding complications are often a result of acquired platelet dysfunction, increased fibrinolysis, and the depletion of coagulation factors [5,7].

Within cardiac surgery, aortic procedures are associated with the highest bleeding complication rate [8]. These complications can significantly worsen patient outcomes, including transfusion-related adverse events, ischemic complications due to a reduction in organ perfusion, and higher mortality [8,9]. Patients with aortic pathology often already present with dysregulated coagulation before surgery as a result of abnormal blood flow in aortic aneurysms or an intimal tear in aortic dissections [10,11]. These disturbances may activate the coagulation cascade, resulting in coagulopathy and, in extreme cases, disseminated intravascular coagulation (DIC) [10,12].

Systematic reviews on coagulation management in cardiac surgery have been published earlier and are included in guidelines [13,14]. However, they do not make a distinction specifically on thoracic aortic surgery and no other guidelines specifically focused on thoracic aortic surgery. This scoping review aims to evaluate current knowledge on intraoperative hemostatic agents in patients undergoing thoracic aortic surgery. We provide an overview of hemostatic agents used and their impact on bleeding and transfusion requirements and identify existing knowledge gaps and directions for future research.

## 2. Materials and Methods

### 2.1. Search Strategy

The Department of Anesthesiology in the Amsterdam UMC, location AMC, performed a search strategy with the help of a medical information specialist (FJ). MEDLINE (PubMed), Embase, and Cochrane Library were searched using predefined search terms from inception until the 2nd of July 2024 (see Supplement S1 for the full search strategy). No filters were used in the search. For the identification of upcoming or ongoing trials, clinicaltrials.gov was searched with the terms “thoracic aortic surgery”, “thoracic aortic aneurysm”, and “acute aortic dissection”, without the use of other filters. This scoping review was conducted in accordance with the Preferred Reporting Items for Systematic Reviews and Meta-Analyses (PRISMA) guidelines [15].

### 2.2. Eligibility Criteria

Thoracic aortic surgery was defined as surgery which required the opening of the thorax, and surgery of the aortic root, ascendens, or arch, with the use of CPB. Surgeries solely on the descending aorta and endovascular surgeries were excluded. Further inclusion criteria were studies written in English, including adult patients, determining the effect of intraoperative administration of blood products, antifibrinolytics, fibrinogen supplementation or coagulation factors, and the effect of these products on bleeding and/or number of blood transfusions. Randomized controlled trials (RCT) and observational trials were included if they comprised of a control group. Studies that primarily focused on the cardiac surgery population but also included thoracic aortic surgery patients were only included if they provided a subgroup analysis. No restrictions were made for the year of publication or size of the study population. Animal studies, in vitro studies, case reports, case series, reviews, editorials, conference notes, and also reports from overlapping or duplicated populations were excluded. Furthermore, non-pharmacological studies for bleeding management (e.g., cell saver, auto-transfusion, and viscoelastic test-guided transfusion algorithms) or topical hemostatic agents were not included in this review.

### 2.3. Selection and Data Collection Process

Both title and abstract screening and full-text screening were performed by two independent reviewers (MH and CB) using Rayyan [16]. Disagreement on article inclusion were resolved by a third reviewer (HH).

The extraction of data was performed by one reviewer (MH). Data consisted of the first author, year of publication, years of inclusion, population, type of study, number of inclusions, methodology, intervention, outcome measurements, and findings of the included reports.

## 3. Results

### 3.1. Included Studies

**Figure 1:**
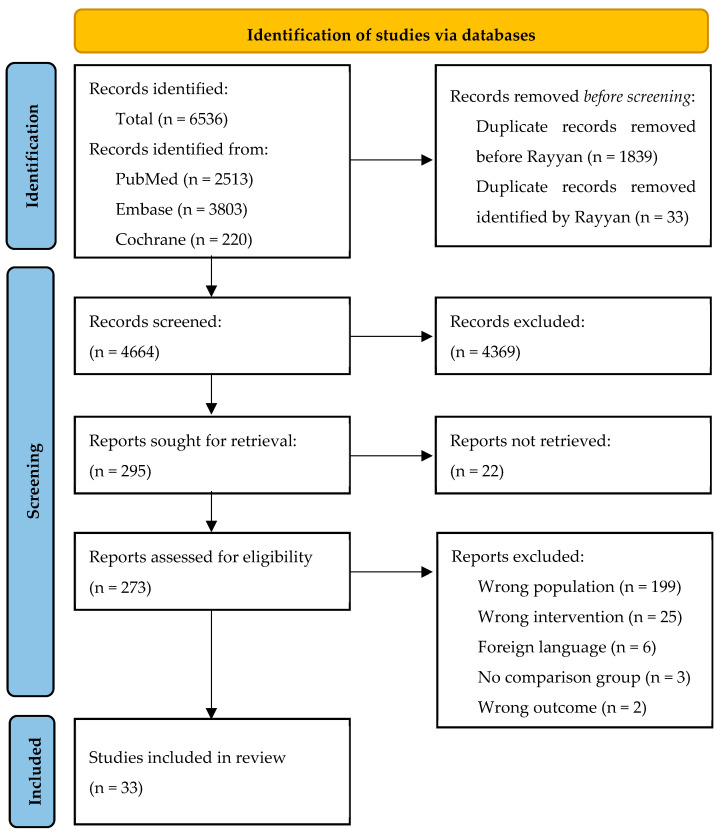
**Flowchart of included studies.** Wrong population included either no cardiac or thoracic aortic surgery or no subgroup analysis of thoracic aortic surgery. Wrong intervention included non-pharmacological interventions (e.g., autotransfusion, cell saver, or acute normal hemodilution) or the effect of transfusion protocols (e.g., ROTEM).

The initial search after deduplication yielded 4664 articles, of which 295 articles were assessed in full-text form, resulting in 33 included articles (Figure 1). Most exclusions in the full text screening were due to absence of subgroup analysis on thoracic aortic surgery.

Of the included studies, three studies were on blood products as an intervention [17,18,19], two studies were on factor eight inhibitor bypassing activity (FEIBA) [20,21] (Table 1), seven studies were on fibrinogen supplementation [22,23,24,25,26,27,28] (Table 2), eight studies were on recombinant factor VIIa (rFVIIa) [29,30,31,32,33,34,35,36] (Table 3), and thirteen studies were on antifibrinolytics [37,38,39,40,41,42,43,44,45,46,47,48,49] (Table 4). No studies were found on other prothrombin complex concentrates (PCCs), factor XIII, or desmopressin in the context of thoracic aortic surgery.

### 3.2. Blood Products

Three studies on blood product transfusion were included [17,18,19] (Table 1).

Stensballe et al. (2018) conducted a single-center, single-blinded RCT comparing OctoplastGP (solvent/detergent-treated, virus-inactivated, pooled human plasma, n = 29) to FFP (n = 28) in dissection patients [17]. While patients and study assessors were blinded, clinicians were not, introducing potential bias. Dosing and transfusion protocols were not clearly reported. OctoplastGP reduced intraoperative blood loss (~600 mL), transfusion requirements, and markers of endothelial injury, with no reported safety concerns.

Wu et al. (2014) retrospectively compared dissection patients who received intraoperative platelet transfusion (n = 74) to those who did not (n = 85) [18]. In-hospital mortality was similar between the groups, but the platelet transfused group had a higher frequency of RBC transfusion in larger volumes, postoperative sternal wound infections, and neurological deficits.

Naeem et al. (2018) performed a non-matched retrospective study on aortic dissection patients [19]. Patients receiving >2 units of RBCs (n = 34) had more post-transfusion infections compared to those receiving 0–2 units RBC (n = 68). Similarly, patients given >1 unit of platelets (n = 28) had higher rates of acute kidney injury and atrial fibrillation than those receiving 0–1 unit (n = 74). Both high-transfusion groups had longer hospital stays, but mortality rates were unchanged.

In conclusion, the impact of blood product transfusion on bleeding outcomes remains unclear, as no studies compared transfusion to placebo or no transfusion while focusing on bleeding control. However, blood product transfusion appears to be associated with increased post-transfusion complications.

### 3.3. Factor Eight Inhibitor Bypassing Activity (FEIBA)

Two studies evaluated the use of FEIBA in aortic dissection surgery [20,21] (Table 1). Sera et al. (2021) conducted a pilot RCT comparing FEIBA (20 IU/kg) to a placebo in six patients per group [20]. The study found numerically lower intraoperative blood products and chest tube drainage, but it was not powered for significance.

Pupovac et al. (2022) performed a retrospective propensity-matched study with 53 patients, administering 500 IU of FEIBA as a salvage measure for persistent bleeding after RBCs, platelets, plasma, and cryoprecipitate [21]. The study showed reduced transfusion requirements in the first 48 h post-surgery, with no difference in thromboembolic complications.

In conclusion, both studies suggest that FEIBA may reduce blood product use in aortic dissection surgery. However, the timing of FEIBA administration was different in these two studies, where it was a rescue therapy in the retrospective study but standard in the pilot RCT.

### 3.4. Fibrinogen Supplementation

Seven studies on fibrinogen supplementation were included [22,23,24,25,26,27,28]. Several additional studies identified in the full-text review phase were excluded as they were re-analyses or post hoc analyses of the REPLACE trials. Only the original REPLACE trials were included. Both the initial single-center REPLACE trial and the subsequent larger multicenter trials were included, as they recruited different patient populations (Table 2).

#### 3.4.1. Fibrinogen Supplementation Compared to Placebo

Three RCTs evaluated fibrinogen supplementation. The multicenter REPLACE trial (2016, 78 patients with fibrinogen vs. 74 with placebo) used ROTEM-guided dosing targeting a FIBTEM A5 of 22 mm, with a mean fibrinogen dose of 6.29 g [22]. Fibrinogen was given if the 5 min post-CPB bleeding mass (measured by sponges and suction canisters) ranged between 60 and 250 g. Unexpectedly, total 24 h blood product use was higher in the fibrinogen group (median five vs. three units), likely due to higher pretreatment bleeding mass, low overall bleeding rates, and variability in adherence to the transfusion algorithm. Notably, 31% of patients had a pretreatment fibrinogen level > 2 g/L. There were no differences in thromboembolic events.

The earlier single-center REPLACE study (2013) with 29 patients receiving fibrinogen concentrate and 32 receiving a placebo showed reduced blood product use with a median fibrinogen dose of 8 g [23].

A more recent single-center RCT by Vlot et al. (2022, 10 patients per group) used the same 5 min bleeding mass as REPLACE. However, fibrinogen dosage was based on weight without applying a target ROTEM value [24]. In their study, 90% (18 patients) had pretreatment fibrinogen levels < 2 g/L. No difference in blood product use was found, though the 5 min bleeding mass was non-significantly reduced by 52% in the fibrinogen group vs. 32% in the placebo group. The study was terminated early due to a slow inclusion rate.

#### 3.4.2. Fibrinogen Supplementation Compared to No Treatment

Three observational studies compared fibrinogen supplementation to no supplementation.

Kikura et al. (2023) conducted a multicenter retrospective study (285 fibrinogen vs. 154 control patients) in both elective and emergency thoracic aortic surgery patients, administering 2–3 g fibrinogen after CPB cessation if fibrinogen < 1.0–1.2 g/L or FIBTEM A10 < 6 mm [25]. No differences were found in major bleeding, re-exploration, or mortality. RBC and FFP transfusions were reduced, while platelet transfusion was increased. A subgroup analysis of patients with fibrinogen levels < 1.5 g/L suggested that fibrinogen reduced major bleeding, despite the study’s transfusion protocol requiring all included patients to have low fibrinogen levels. Li et al. (2022) studied 105 aortic dissection patients receiving 2 g fibrinogen preoperatively and 54 controls, finding reduced intraoperative RBC transfusion, chest tube drainage, and shorter ventilation and hospital stays [26]. Guan et al. (2023) compared 54 aortic dissection patients receiving 25–50 mg/kg fibrinogen after protamine reversal to 30 controls, showing reduced blood transfusions and chest tube drainage [27].

#### 3.4.3. Fibrinogen Supplementation Compared to FFP

Yamamoto et al. (2014) compared fibrinogen concentrate (n = 25, 3–5 g) to FFP (n = 24) in patients with fibrinogen < 1.5 g/L at the end of CPB, including both elective and dissection cases. Fibrinogen concentrate significantly reduced intraoperative blood loss and led to lower RBC, FFP, and platelet transfusion requirements [28].

In conclusion, the evidence on fibrinogen supplementation in thoracic aortic surgery remains inconclusive. The multicenter REPLACE RCT showed a negative effect (more blood transfusion), while the single-center REPLACE RCT and a smaller single-center RCT suggested positive effects (less blood transfusion). Three retrospective studies indicated benefits of fibrinogen supplementation with no increased thromboembolic complications, with one showing superiority over FFP. Differences in pretreatment fibrinogen levels and patient selection may explain the variability in results.

### 3.5. Recombinant Factor VIIa (rFVIIa)

Eight studies on rFVIIa were included [29,30,31,32,33,34,35,36] (Table 3). Yan et al. (2014) conducted a non-randomized RCT in aortic dissection patients, where treatment decisions were determined by the patients’ family, with 25 patients in the intervention group receiving rFVIIa (2.4 mg) plus platelet transfusion and 46 patients receiving conventional therapy (RBCs, FFP, platelets, and cryoprecipitate) after the cessation of CPB [29]. The intervention reduced intraoperative transfusion and postoperative platelet use, but no differences were observed in postoperative blood loss or adverse events.

Six observational studies in dissection patients and one observational study on thoracic aortic patients including dissections were included: Zindovic et al. (2017) performed a multicenter propensity-matched study on 120 dissection patients, but rFVIIa dosage and transfusion protocols were not clearly defined [30]. The rFVIIa group received more blood products, had more chest tube drainage, and had more re-explorations due to bleeding. Keyoumu et al. (2024) found that rFVIIa reduced chest tube drainage and RBC/FFP transfusions but was associated with more thromboembolic complications and higher mortality [31]. Andersen et al. (2012) reported reduced postoperative blood product use, with no difference in complications in the rFVIIa group [32]. Goksedef et al. (2012) found that rFVIIa reduced postoperative chest tube drainage, transfusion, and reoperations [33]. Ise et al. (2022) reported no difference in blood loss or transfusion between the rFVIIa and no rFVIIa group [34]. Tritapepe et al. (2007) showed that rFVIIa reduced postoperative blood loss but required more blood products [35]. Lastly, Hang et al. (2021) compared rFVIIa patient controls in both elective and dissection patients and found was no difference in postoperative transfusion, chest tube drainage, or complications, with a trend toward higher reoperation rates [36].

In conclusion, the studies on the efficacy of rFVIIa present conflicting results. Bias appears to be present, as the rFVIIa groups often show higher intraoperative blood product use, even after matching. The non-randomized prospective trial has limitations, such as unclear descriptions of the transfusion protocol in the usual care group.

### 3.6. Antifibrinolytics

#### 3.6.1. Tranexamic Acid (TXA) Versus Placebo or No TXA

In an RCT, Casati et al. (2002) compared TXA (1 g bolus before incision, 400 mg/hour infusion) to a placebo in elective thoracic aortic surgery [37] (Table 4). In 29 patients per group, TXA reduced perioperative red blood cell (RBC) transfusion and postoperative blood loss. The incidence of excessive bleeding (>600 mL/24 h) was lower in the TXA group, with no difference in thromboembolic complications. Similarly, Ahn et al. (2015) compared a continuous TXA infusion (16 mg/kg/h) to no TXA in 26 dissection patients, demonstrating reduced transfusion requirements and chest tube drainage, again without increased thromboembolic complications [38].

#### 3.6.2. TXA Versus Epsilon-Aminocaproic Acid (EACA) or Aprotinin

In an RCT comparing TXA to EACA, Makhija et al. (2013) included 61 patients undergoing thoracic aortic surgery (TXA: 31 patients, 10 mg/kg bolus, 1 mg/kg/h infusion and EACA: 30 patients, 50 mg/kg bolus, 25 mg/kg/h infusion) [39]. No significant differences in blood loss or transfusion requirements were found, though TXA showed a non-significant trend towards more seizures.

Four retrospective studies compared TXA to aprotinin. Reidy et al. (2024) matched 49 pairs of dissection patients receiving TXA (1 g bolus, 500 mg/h infusion) or aprotinin (2 million unit bolus, 500,000 units/h infusion) [40]. No differences were found in transfusions, drainage, mortality, or reoperation. Nicolau-Raducu et al. (2010) showed a trend toward lower blood product use in the aprotinin group but more renal dysfunction [41]. Sniecinski et al. (2010) found that TXA was associated with increased fresh frozen plasma (FFP) and cryoprecipitate transfusions, with a non-significant higher incidence of seizures [42]. Chivasso et al. (2018) analyzed 107 matched patients, comparing TXA to aprotinin, with no differences in major bleeding or reoperation, though FFP use was higher in the aprotinin group [43]. Sedrakyan et al. (2006) found that aprotinin (84 matched pairs) reduced platelet transfusions and chest tube drainage, but the timeframe of the outcomes was unclear [44].

#### 3.6.3. Aprotinin Versus Placebo or No Aprotinin

Numerous studies on aprotinin use in cardiac surgery appeared following its market withdrawal after the BART trial [50] due to an increased risk of death in the trial, though debate persists within the scientific community regarding the appropriateness of this decision. In this review, we only report the studies on aprotinin in patients undergoing thoracic aortic surgery. In a placebo-controlled trial, Ehrlich et al. (1998) randomized 25 patients to receive 140 mg aprotinin and 25 patients to receive a placebo (saline). While no difference in renal function (primary outcome) was found, aprotinin reduced blood product use and chest tube drainage [45]. Westaby et al. (1994) compared 53 aprotinin patients (100 mg bolus, 25 mg/h infusion) to 29 controls and found no reduction in chest tube drainage; Interestingly, they reported increased blood loss in aprotinin-treated patients after 1989, coinciding with the introduction of hypothermic circulatory arrest, though blood loss assessment methods were unclear [46]. In contrast, Parolari et al. (1997) found reduced blood product use and blood loss in patients receiving 700 mg aprotinin (18 vs. 21 controls) but noted a trend toward more neurological deficits and complications [47]. Similarly, Seigne et al. (2000) showed reduced RBC, FFP, and platelet use with aprotinin (5 mg bolus, 5 mg/h infusion, 9 patients vs. 10 controls) without an effect on chest tube drainage [48].

#### 3.6.4. Aprotinin Versus EACA

A single retrospective study by Eaton et al. (1998) compared aprotinin (n = 29, 100 mg bolus, 25 mg/h infusion) to EACA (n = 19, 5–40 g bolus, 25 mg/h infusion) in thoracic aortic surgery [49]. Five patients received both agents. No differences were found in RBC transfusion or chest tube drainage at six and twelve hours. Intraoperatively, the EACA group received more platelet transfusions and had a higher incidence of acute renal failure.

In conclusion, two small RCTs (each with <30 patients per group) comparing antifibrinolytics to placebo or no treatment, one with TXA and one with aprotinin, demonstrated reduced transfusion requirements and chest tube drainage without an increase in adverse events. The available retrospective studies provided inconsistent results, as follows: (1) three studies comparing aprotinin to no treatment showed mixed findings, with two suggesting a lack of benefit or potential harm, while one reported a positive effect (of note, the aprotinin studies were all published in the years 1994–2000), (2) one study comparing TXA to no treatment reported a positive effect for TXA, and (3) comparative studies between different types of antifibrinolytics gave no consistent signal favoring one over the other. The current evidence base is limited by heterogeneous study designs, varying antifibrinolytic dosages, and inconsistent outcome reporting, making a meta-analysis not feasible.

### 3.7. Upcoming or Ongoing Trials

Clinicaltrials.gov was searched on the 12th of March 2025 to identify upcoming or ongoing trials with the search term ‘thoracic aortic surgery’, resulting in 161 hits. No trials were found on intraoperative systemic hemostatic interventions. The search term ‘thoracic aortic aneurysm’ produced 388 hits. No relevant trials were found. The search term ‘acute aortic dissection’ yielded 396 results, of which one was a relevant non-randomized prospective trial on fibrinogen concentration (NCT02542306); however, this had not been updated since 2015 and no linked article could be found.

## 4. Discussion

There is limited evidence on hemostatic agents in thoracic aortic surgery patients. Smaller studies suggest potential for routine use of antifibrinolytics, FEIBA, and possibly fibrinogen supplementation, but only in bleeding patients with hypofibrinogenemia. The evidence on rFVIIa is conflicting. No studies on other prothrombin complex concentrates, FXIII, or desmopressin were found. Further high-quality, ideally randomized controlled trials, specifically focused on thoracic aortic procedures, are necessary to identify optimal coagulation management in these vulnerable patients.

### 4.1. Unique Challenges in Thoracic Aortic Surgery

Thoracic aortic surgery presents distinct hemostatic challenges compared to general cardiac surgery. Excessive bleeding occurs at a significantly higher rate (~15%) compared to coronary artery bypass grafting (CABG) (~5%) [8]. This is due to the complexity of the procedure, which often involves deep hypothermic circulatory arrest, prolonged aortic clamping, and extended CPB—all of which impact coagulation [51]. Furthermore, pre-existing coagulation abnormalities in aortic aneurysms and aortic dissections, as shown by elevated D-dimer levels [52,53] and coagulation factor consumption [10,11,54,55], may increase bleeding risk. Given these higher event rates, general cardiac surgery studies with lower event rates may underestimate the effect of interventions in this population. A further consideration is the difference between elective and acute aortic procedures. Elective surgeries, such as planned aortic aneurysm repairs, allow for preoperative optimization of coagulation status and blood conservation strategies. In contrast, acute aortic surgeries, particularly those for dissections, are performed under emergency conditions with high transfusion requirements, coagulopathy, and greater reliance on hemostatic interventions. This distinction is not always addressed in existing studies but may influence both clinical decision making and research outcomes.

### 4.2. Comparison with Guidelines

The 2024 European Association for Cardio-Thoracic Surgery (EACTS) and European Association of Cardiothoracic Anaesthesiology and Intensive Care (EACTAIC) guidelines strongly recommend antifibrinolytics, particularly TXA, to reduce bleeding, transfusions, and reoperations [13,14]. The guideline does not specifically make a distinction between cardiac surgery and thoracic aortic surgery. Of note, evidence for EACA is limited, and aprotinin, which may be effective, carries safety concerns. Our review aligns with these conclusions, finding no significant differences between TXA, EACA, and aprotinin. Additionally, a recent meta-analysis not included in the guideline further supports the benefits of TXA [56].

For fibrinogen supplementation, the guideline advises its use only in patients with confirmed hypofibrinogenemia, which aligns with our findings. While the REPLACE trials showed no overall beneficial effect, observational studies indicated benefits in patients with low fibrinogen levels. The guideline also favors PCC over FFP for coagulopathic bleeding, a recommendation supported by recent findings [57]. However, the Society of Cardiovascular Anesthesiologists (SCA) guideline does not provide a strong recommendation for routine use of PCC [14]. We found no studies for PCC in thoracic aortic surgery, apart from FEIBA, which showed promising results but requires further study.

Regarding rFVIIa, the guidelines advise against prophylactic use but allows for its use in refractory bleeding. Based on our review no definitive conclusion on rFVIIa use in thoracic aortic surgery could be drawn, as studies showed conflicting results regarding mortality and thromboembolic events.

For desmopressin and FXIII, no thoracic aortic surgery-specific studies were found. The EACTS-EACTA guidelines advise against routine use of FXIII and limits desmopressin to select cases. Regarding blood product use, both guidelines recommend restrictive RBC transfusion thresholds of ≤7.5 g/L.

While our findings largely align with current guidelines regarding antifibrinolytics and fibrinogen supplementation, specific data for thoracic aortic surgery remain sparse, and many recommendations in the guidelines are extrapolated from general cardiac surgery. Larger, targeted trials are needed to refine guidelines for this high-risk population.

### 4.3. Knowledge Gaps and Future Research

The EACTS/EACTAIC guidelines identify knowledge gaps in intraoperative procoagulant products, including TXA timing and dose, the role of aprotinin, and the impact of decreased FXIII activity. We argue that larger RCTs are needed specifically for thoracic aortic surgery, as these patients are at higher risk for bleeding and complications.

One key research priority is to stratify patients before study inclusion, ensuring that interventions are tested in those with certain documented coagulation profiles. For example, while the REPLACE trials on fibrinogen showed no benefit overall, a trial focusing solely on patients with hypofibrinogenemia (<1.5 g/L) could yield more meaningful results. Similarly, further studies on FEIBA, particularly in a randomized setting, are warranted.

Another challenge is the heterogeneity in study designs, including differences in dosing regimens, transfusion protocols, and outcome measures. Standardizing outcome reporting, particularly regarding bleeding assessment, is essential. Many studies lack clarity in defining blood loss, as factors, like cell saver blood, hemodilution, and gauze-absorbed bleeding, complicate accurate measurement. A promising measure could be the Universal Definition of Perioperative Bleeding (UDPB) score [58], which includes transfusions, reoperations, and chest tube drainage and is, among others, one of the recommendations in a recent consensus statement from 2025 [59]. This score has been validated for cardiac surgery and correlates with mortality. However, its application to thoracic aortic surgery remains untested and could be a valuable research direction.

## 5. Conclusions

Patients undergoing thoracic aortic surgery face distinct challenges in coagulation management that necessitate dedicated studies rather than extrapolation from general cardiac surgery. The current evidence base is limited, but small studies suggest potential benefits for routine antifibrinolytic use, FEIBA, and fibrinogen supplementation in bleeding patients with hypofibrinogenemia. Evidence for rFVIIa remains conflicting, and no studies on other PCCs, FXIII, or desmopressin were identified. Larger randomized trials with standardized outcome measures are required to refine hemostatic strategies for this high-risk population.

## Figures and Tables

**Table 1:** Summary of the included studies on blood products (n = 4) and FEIBA (n = 2).

Author, Year, Country	Type of Study	Population	Intervention	Number of Included Patients	Findings	Effect of Intervention
**Blood products**						
Stensballe, 2018, Denmark [17]	Single-blinded RCT (clinicians not blinded)	Acute type A dissections	OctoplastLG vs. standard FFP Trigger/timing: unclear Dose: unclear	29/28	OctoplastLG reduced endothelial injury. Decreased 24 h total transfusion and platelet transfusion volume and goal-directed use of procoagulants. No safety concerns were raised.	FFP: 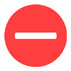
Wu, 2014, China [18]	Retrospective	Acute type A dissections	Platelet transfusion vs. no platelet transfusion Trigger/timing: intraoperative, but otherwise unclear Dose: unclear	74/85	In-hospital mortality was similar. Postoperative sternal wound infection, neurological deficits, postoperative transfusion volume and percentage of RBC was increased in the platelet-transfused group.	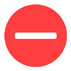
Naeem, 2018, USA [19]	Retrospective	Acute type A dissections	0–2 vs. >2 RBC, and 0–1 platelet unit vs. >1 platelet unit Trigger/timing: unclear Dose: as per stratification	68/34	Rate of postoperative infections higher in patients receiving >2 units of RBC and independent risk factor. AKI and atrial fibrillation more frequent in patients receiving >1 unit of platelets and independent risk factor. Hospital stay longer in patients who received >2 units RBC or >1 unit platelets. No significant differences in mortality.	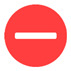
**FEIBA vs. placebo or no FEIBA**						
Sera, 2021, USA [20]	Double-blind RCT	Elective thoracic aortic surgery	FEIBA vs. placebo (saline) Trigger/timing: post-CPB, standard, regardless of bleeding or not Dose: 20 IU/kg concentration of 40 IU/mL, at a rate of 0.5 mL/kg via infusion over 10 min	6/6	Pilot trial. DHCA longer in FEIBA group. Substantial variability but no difference in post-randomization blood products transfused. No difference in chest tube drainage, duration of intubation and hospital length of stay. Two patients (both in FEIBA group) experienced postoperative cerebrovascular events and died, considered not to be related to FEIBA.	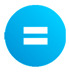
Pupovac, 2022, USA [21]	Retrospective, matched (not by intraoperative transfusion)	Acute type A dissections	FEIBA vs. no FEIBA Trigger/timing: salvage therapy during surgery Dose: 500 units, repeated if necessary	Before matching: 112/119 After matching: 53/53	Intraoperatively, no difference in blood product use. After surgery in the first 48 h, decreased blood product use in FEIBA group A greater proportion of the no FEIBA group received factor VIIa. No differences in incidences of thromboembolic complications	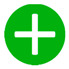

RCT = randomized controlled trial; RBC = red blood cell; FFP = fresh frozen plasma; CPB = cardiopulmonary bypass; FEIBA = factor eight inhibitor bypassing activity; AKI = acute kidney injury; USA = United States of America.

**Table 2:** Summary of included studies on fibrinogen suppletion (n = 7).

Author, Year, Country	Type of Study	Population	Intervention	Number of Included Patients	Findings	Effect of Intervention
**Fibrinogen suppletion versus placebo or no fibrinogen suppletion**						
Rahe-Meyer, 2016, Worldwide REPLACE-II [22]	Double-blind RCT, multicenter	Open surgical procedures with CPB on any part of the aorta, provided a 5 min bleeding rate of 60–250 g. (emergency surgery excluded, thoracoabdominal included)	Fibrinogen concentrate vs. placebo (saline) Trigger: 60–250 g 5-min bleeding post-CPB Target: A10 FIBTEM of 22 mm Mean dose: 6 g	78/74	Fibrinogen group received more units of total blood products in the first 24 h compared to the placebo (5 vs. 3 units). FFP transfusions higher for the fibrinogen group but no significant differences in platelet or RBC transfusions. Placebo group had a higher percentage of patients avoiding transfusion. The initial 5 min bleeding mass was higher in the fibrinogen group. Furthermore, 31% of the total cohort had a pretreatment fibrinogen level of >2 g/L. Thromboembolic events occurred in 7.7% of the fibrinogen group and 13.5% of the placebo group.	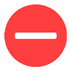
Rahe-Meyer, 2013, Germany REPLACE-I [23]	Double-blind RCT, single-center	Open surgical procedures with CPB on any part of the aorta, provided a 5 min bleeding rate of 60–250 g. (emergency surgery excluded, thoracoabdominal included)	Fibrinogen concentrate vs. placebo (saline) Trigger: 60–250 g 5-min bleeding post-CPB Target: A10 FIBTEM of 22 mm Mean dose: 8 g	29/32	Comparable in characteristics and 5 min bleeding mass. Fibrinogen group received fewer units of total blood products in the first 24 h after study medication. Total transfusion avoidance was achieved in 45% in fibrinogen group, while all placebo patients were transfused. No safety concerns were noted. Incidences of thromboembolic patients did not differ (one in fibrinogen group, two in placebo group).	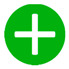
Vlot, 2022, Netherlands [24]	Double-blind RCT	Aortic arch surgery, provided a 5 min bleeding mass of 60–250 g Excluded: preoperative fibrinogen concentration < 1 g/L	Fibrinogen concentrate vs. placebo (saline) Trigger: 60–250 g 5-min bleeding post-CPB Dose: 4 g for <70 kg; 6 g for 70–90 kg; 8 g for >90 kg	10/10	There was no difference in allogeneic blood transfusion in the first 24 h after study medication. Before treatment, 90% of patients had a fibrinogen concentration < 2 g/L. The 5 min bleeding mass decreased by 52% in the fibrinogen group and 32% in the placebo group. Thromboembolic events were reported in one patient per group.	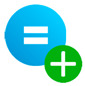
Kikura, 2023, Japan [25]	Retrospective, multicenter	Thoracic aortic surgery (including emergency)	Cryoprecipitate or fibrinogen concentrate vs. no product Trigger: fibrinogen < 1.0–1.2 g/L post-CPB or FIBTEM A10 < 6 mm during rewarming Dose: 2–3 g	285/154	No difference in incidence of major bleeding, re-exploration, or mortality. n patients with fibrinogen replacement and fibrinogen level < 1.5 g/L and a lower incidence of major bleeding compared to control. Decreased use of RBC and FFP, but increased use of platelet transfusion intraoperatively. Data on thromboembolic events were not reported.	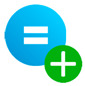
Li, 2022, China [26]	Retrospective	Acute type A dissections	Fibrinogen concentrate vs. no fibrinogen concentrate Trigger: standard preoperatively Dose: 2 g	105/54	Reduced intraoperative blood loss and RBC transfusion. Reduced postoperative chest tube drainage. Data on thromboembolic events were not reported.	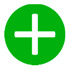
Guan, 2023, China [27]	Retrospective analysis of a prospective database	Acute type A dissections	Fibrinogen concentrate vs. no fibrinogen concentrate Trigger: fibrinogen < 1.5 g/L after protamine Target: fibrinogen > 2.0 g/L Dose: initially 25–50 mg/kg (~2–5 g for 70–90 kg)	54/30	Blood product use (RBC, FFP, platelets) decreased from the moment of infusion until the 5th postoperative day. Total transfusion avoidance was achieved in 17% of fibrinogen group, while all patients in control group received transfusions. The 24 h and 48 h postoperative drainage were lower in the fibrinogen group, though not maintained in total drainage volumes. Thromboembolic events were reported in none of the no fibrinogen group patietns and in two of the fibrinogen group patients.	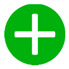
**Fibrinogen concentrate versus FFP**						
Yamamoto, 2014, Japan [28]	Retrospective, age-matched	Elective thoracic aortic surgery with fib < 1.5 at the end of CPB	Fibrinogen concentrate vs. FFP Trigger: fibrinogen < 1.5 g/L after CPB Target: fibrinogen > 2.0 g/L Dose: 3–5 g	25/24	In the fibrinogen group, the average volume of intraoperative blood loss decreased by 64%, while the average number of transfusion units was reduced by 56% in RBC, 61% in FFP, and 55% in platelets compared to cases where only FFP was administered. Data on thromboembolic events were not reported.	Fibrinogen concentrate: 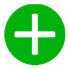

RCT = randomized controlled trial; CPB = cardiopulmonary bypass; RBC = red blood cell; FFP = fresh frozen plasma.

**Table 3:** Summary of included studies on rFVIIa (n = 8).

Author, Year, Country	Type of Study	Population	Intervention	Number of Included Patients	Findings	Effect of Intervention
Yan, 2014, China [29]	Non-randomized prospective trial (family of patients decided allocation)	Acute type A dissections	Platelets + rFVIIa vs. conventional (RBCs, FFPs, platelets, cryoprecipitate) Trigger/timing: standard, during surgery Dose: 3 units of platelets, 2.4 mg of rFVIIa	25/46	Decreased intraoperative RBC, FFP, platelets and cryoprecipitate use and postoperative platelet use. Less time to sternal closure. Blood loss similar at 1, 6 and 12 h after surgery. No difference in serious adverse events. One patient in each group experienced a stroke.	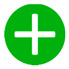
Zindovic, 2017, Finland [30]	Retrospective, matched (not by intraoperative transfusion), multicenter	Acute type A dissections	rFVIIa vs. no rFVIIa Trigger/timing: salvage therapy during surgery or in the ICU Dose: unknown	Before matching: 590/171 After matching: 120/120	The rFVIIa group received more RBCs, platelets and FFP. This group underwent re-exploration for bleeding more often and had greater 24 h chest tube drainage. No difference in mortality, stroke, or renal replacement therapy.	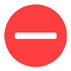
Keyoumu, 2024, China [31]	Unclear (as it includes dissections, presumably retrospective), no matching described	Acute type A dissections (onset < 12 h, <65 year)	rFVIIa vs. no rFVIIa Trigger/timing: salvage therapy during surgery Dose: 100 µg/kg	60/60	Duration of CPB was longer in the rFVIIa group. Decreased chest tube drainage and transfusion volumes postoperatively, but higher mortality and thromboembolic events (unclear which type of thrombosis) in the rFVIIa group.	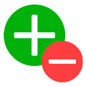
Andersen, 2012, USA [32]	Retrospective, matched (e.g., intraoperative transfusion)	Thoracic aortic surgery (including emergency) Excluded: patients who received 60 μg/kg or more of rFVIIa	Low dose rFVIIa vs. no rFVIIa Trigger/timing: salvage therapy during surgery Dose: initial dose of <60 μg/kg	44/44 matched	Improved coagulation indicators (INR, aPTT) and decreased postoperative transfusions. No differences in postoperative complications (including thromboembolic complications). Two patients experience an embolic stroke in the intervention group, compared to none in the control group.	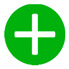
Goksedef, 2012, USA [33]	Retrospective, matched (not by intraoperative transfusion)	Thoracic aortic surgery (aneurysm database, also 1 dissection included)	rFVIIa vs. no rFVIIa Trigger/timing: salvage therapy during surgery Dose: 1 to 2 mg (10–30 μg/kg), repeated once if bleeding persisted.	Before matching: 37/339 After matching: 29/29	Decreased chest tube drainage, decreased postoperative transfusion of RBC and FFP, decreased incidence of resurgical intervention due to bleeding. No differences in thromboembolic events or mortality. In the intervention group, one patient had a stroke and one patient had a transient ischemic attack (TIA), compared to two strokes and one TIA in the control group.	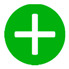
Ise, 2022, Japan [34]	Retrospective, matched (not by intraoperative transfusion)	Thoracic aortic surgery (including dissections)	rFVIIa vs. no rFVIIa Trigger/timing: salvage therapy during surgery Dose: 5, 2 or 1 mg	Before matching: 42/102 After matching: 29/29	More blood loss and transfusion and fibrinogen concentrate intraoperatively. Postoperative bleeding and amount of transfusion significantly higher in the rFVIIa group in the unmatched cohort, but not in the matched cohort. No difference in mortality and thrombosis-related AESs. One patient per group experienced a stroke.	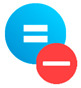
Tritapepe, 2007, Italy [35]	Retrospective, matched (not by intraoperative transfusion)	Acute type A dissections	rFVIIa vs. no rFVIIa Trigger/timing: on the ICU, if surgical cause was excluded and if bleeding exceeded 150 mL/h Dose: 70 μg/kg, repeated if no reduction of bleeding to <150 mL/h.	Before matching: 23/150 After matching: 23/23	Significant reduction in hourly blood loss was found 1 h after rFVIIa administration, as opposed to no difference in hourly blood loss in the control group. Treated patients received larger amounts of blood products. Blood product usage after rFVIIa was lower than before administration. No effect of rFVIIa on adverse events and mortality. One patient in the intervention group experienced a stroke, while none did in the control group.	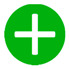
Hang, 2021, USA [36]	Retrospective	Thoracic aortic surgery (including dissections)	rFVIIa vs. no rFVIIa Trigger/timing: salvage therapy during surgery Dose: range of 20–90 µg/kg at the physicians discretion.	20/39	More intraoperative blood products transfused the rFVIIa group. Chest tube drainage in the first 24 h was similar. No difference in total additional blood products and the percentage of patients who required them in the ICU. There was a trend towards higher rate of reoperation for bleeding in rFVIIa group. Postoperative thromboembolic events and mortality were similar. Two patients in the intervention group and five patients in the control group experienced a stroke.	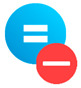

rFVIIa = recombinant activated factor VII; RBC = red blood cell; FFP = fresh frozen plasma; CPB = cardiopulmonary bypass; ICU = intensive care unit; USA = United States of America.

**Table 4:** Summary of included studies on antifibrinolytics (n = 13).

Author, Year, Country	Type of Study	Population	Intervention	Number of Included Patients	Findings	Effect of Intervention
**TXA vs. placebo or no TXA**						
Casati, 2002, Italy [37]	Double-blind RCT	Elective thoracic aortic surgery	TXA vs. placebo Dose: 1 g bolus, 400 mg/h infusion	29/29	Lower incidence of excessive bleeding (>600 mL chest tube drainage/24 h). Less blood loss in the first 24h and decreased perioperative RBC use. No difference in complications. One patient in the TXA group experienced a stroke, while none did in the placebo group. One patient per group experienced myocardial infarctions.	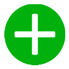
Ahn, 2015, Japan [38]	Retrospective	Acute type A dissections	TXA vs. no TXA Dose: no bolus, 16 mg/kg/h infusion, max 1000 mg/h	26/29	Decreased intraoperative and postoperative blood product use (RBC, FFP, platelets). Decreased chest tube drainage. No difference in complications. Five patients in the TXA group experienced a stroke, while seven did in the control group. One patient had a seizure in the TXA group, while none did in the control group.	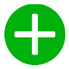
**TXA versus EACA or aprotinin**						
Makhija, 2013, India [39]	RCT	Thoracic aortic surgery (including emergency surgery)	EACA vs. TXA Dose: TXA 10 mg/kg bolus, 1 mg/kg/h infusion. EACA 50 mg/kg bolus, 25 mg/kg/h infusion	30/31	No difference in intra- or postoperative blood product use. No difference in chest tube drainage. More renal injury in the EACA group. Possibly more seizures in the TXA group. One patient had a seizure in the EACA group, while three did in the TXA group. One patient per group experienced a stroke.	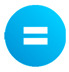
Reidy, 2024, UK [40]	Retrospective, matched	Acute type A dissections	Aprotinin vs. TXA Dose: TXA 1 g bolus, 500 mg/h infusion. Aprotinin 2 million unit bolus, 500,000 units/h infusion	Before matching 82/149 After matching 49/49	No differences in the amount of blood products transfused, 0chest tube drainage, mortality, return to theater, or CVVH (both in matched and unmatched cohorts). In the matched cohort, there were more patients with an open chest in the aprotinin group (2 vs. 0) but not in the unmatched cohort. After matching, 7 patients in the aprotinin group versus 9 patients in the TXA group experienced a stroke.	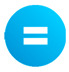
Nicolau-Raducu, 2010, USA [41]	Retrospective	Thoracic aortic surgery with DHCA	Aprotonin vs. TXA Dose: TXA 30 mg/kg bolus, 15 mg/kg/h infusion. Aprotinin 2 million unit bolus, 500,000 units/h infusion	48/36	Aprotinin group: longer CPB time. Trend toward fewer intraoperative blood product transfusions, but not statistically significant. No difference in chest tube drainage. More renal dysfunction, though not identifiable as risk factor in regression. Postoperative complications were similar between groups. Eight patients in the aprotinin group versus seven patients in the TXA group experienced a stroke.	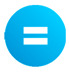
Sniecinski, 2010, USA [42]	Retrospective	Thoracic aortic surgery with DHCA (including emergency surgery)	Aprotinin vs. TXA Dose: TXA 2 g bolus, 500 mg/h infusion. Aprotinin 2 million unit bolus, 500,000 units/h infusion	82/78	Increased use of RBC, FFP, platelets, cryoprecipitate, and rFVIIa in TXA group. Trend towards more seizures in the TXA group but not significant (5 patients in the TXA group versus none in the aprotinin group). No difference in other complications. Two patients per group experienced a stroke.	Aprotinin: 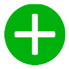 TXA: 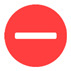
Chivasso, 2018, UK [43]	Retrospective, matched	Thoracic aortic surgery (including dissections)	Aprotinin vs. no aprotinin (they state) but actually vs. TXA Dose: TXA 15 mg/kg bolus, 4.5 mg/kg/h infusion. Aprotinin 2 million unit bolus, 420,000 units/h infusion (70 mg/h)	107/425 before matching 107/107 matched	Higher use of FFP in both matched and unmatched cohorts in the aprotinin group. No difference in other blood products, postoperative bleeding or complications. Five patients in the aprotinin group experienced a stroke, while seven did in the no aprotinin (TXA) group.	Aprotinin: 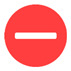
Sedrakyan, 2006, USA [44]	Retrospective, matched	Thoracic aortic surgery (aneurysms, dissections, ulcers, and hematomas)	Aprotinin vs. no aprotinin (but actually compared to EACA and TXA) Dose aprotinin: 2 million unit bolus, 500,000 units/h infusion	No before matching available After matching 84/84	Difference at baseline after matching: controls were more likely to receive antifibrinolytics (EACA or TXA), essentially comparing aprotinin to an alternative blood loss reduction strategy that included antifibrinolytic therapy > 50% of the time. Aprotinin reduced the intraoperative amount of platelet transfusion and resulted in less chest tube drainage in the first 24 h. There were no associations with thromboembolic complications.	Aprotinin: 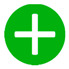
**Aprotinin versus placebo or no aprotinin**						
Ehrlich, 1998, Austria [45]	Double-blind RCT	Elective thoracic aortic surgery (including chronic dissections)	Aprotinin vs. placebo (saline) Dose aprotinin: 1 million unit bolus before CPB	25/25	Aprotinin decreased chest tube output in the first 24 h and decreased transfusion requirements (RBC, FFP, platelets, cryoprecipitate). One patient in the no aprotinin group experienced a stroke, while none did in the aprotinin group.	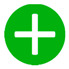
Westaby, 1994, UK [46]	Both retrospective and prospective	Acute type A dissections	Aprotinin vs. no aprotinin Dose aprotinin: 2 million unit bolus, 500,000 units/h infusion	53/29	No reduction in overall blood loss and transfusion requirements for aprotinin patients. After the introduction of hypothermic circulatory arrest (1989), aprotinin patients consistently experienced more blood loss. There were 5 deaths per group. In the aprotinin group, two of those were due to myocardial infarction and one due to pulmonary embolisms, in the control group none were due to thromboembolic complications.	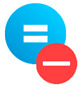
Parolari, 1997, Italy [47]	Retrospective	Thoracic aortic surgery with DHCA (including dissections)	Aprotinin vs. no aprotinin Dose: 700 mg.	18/21	No difference in postoperative blood loss, blood product use, re-exploration, mortality or complications. However, the aprotinin group showed a higher trend towards neurological deficit and a more complicated postoperative course.	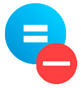
Seigne, 2000, USA [48]	Retrospective	Elective or urgent thoracic aortic surgery with DHCA (all Bentalls)	Aprotinin vs. no aprotinin Dose aprotinin: 1 million unit bolus, 100,000 units/h infusion	9/10	Aprotinin decreased RBC, FFP, and platelet use intraoperatively and FFP postoperatively. No difference in chest tube drainage. Two patients in the non-aprotinin group developed seizures (none in the aprotinin group), while one patient experienced a stroke in the aprotinin group (none in the non-aprotinin group).	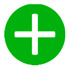
**Aprotinin versus EACA**						
Eaton, 1998, USA [49]	Retrospective	Thoracic aortic surgery (including dissections)	Aprotinin vs. EACA Dose: aprotinin 2 million unit bolus, 500,000 units/h infusion. EACA 5–40 g with a bolus/infusion scheme	29/19	No difference in chest tube output or postoperative transfusion. Possibly more renal failure in the EACA group. No differences in other complications. Three patients in the aprotinin group and five patients in the EACA group experienced strokes.	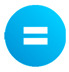

RCT = randomized controlled trial; TXA = tranexamic acid; EACA = epsilon-aminocaproic Acid; DHCA = deep hypothermic circulatory arrest; CPB = cardiopulmonary bypass; RBC = red blood cell; FFP = fresh frozen plasma; UK = United Kingdom; USA = United States of America.

## Data Availability

The search strategy can be found in the Supplementary File.

## Supplementary Materials

The following supporting information can be downloaded at: https://www.mdpi.com/article/10.3390/jcm14114001/s1, S1: Search Strategy.

## Abbreviations

The following abbreviations are used in this manuscript:

EACA	Epsilon-aminocaproic acid
FEIBA	Factor eight inhibitor bypassing activity
FFP	Fresh frozen plasma
PCC	Prothrombin complex concentrate
rFVIIa	Recombinant factor VIIa
RCT	Randomized controlled trial
TXA	Tranexamic acid
