# Supplementary material for: Intraoperative Hemostatic Agents in Thoracic Aortic Surgery—A Scoping Review
Source: J Clin Med. 2025 Jun 5;14(11):4001. doi: 10.3390/jcm14114001 (PMC12156886; doi:10.3390/jcm14114001)
Supplement: Supplementary file 1 [file jcm-14-04001-s001.zip › jcm-3658149-supplementary.pdf]

Additional file

Intraoperative Hemostatic Agents in Thoracic Aortic Surgery—A Scoping Review

Maite M. T. van Haeren [1,2], Caitlin Bozic [1], Jennifer S. Breel [1], Susanne Eberl [1], Faridi S. Jamaludin [3], Denise P. Veelo [1], Marcella C. A. Müller [2], Alexander P. J. Vlaar [2] and Henning Hermanns [1,*]

[1] Department of Anesthesiology, Amsterdam University Medical Centers, 1105 AZ Amsterdam, The Netherlands
[2] Department of Intensive Care, Amsterdam University Medical Centers, 1105 AZ Amsterdam, The Netherlands; m.c.muller@amsterdamumc.nl (M.C.A.M.)
[3] Medical Library, Amsterdam University Medical Centers, Location University of Amsterdam, 1105 AZ Amsterdam, The Netherlands; f.s.vanetten@amsterdamumc.nl
* Correspondence: h.hermanns@amsterdamumc.nl; Tel.: +31-20-5662099

Supplement S1: Search strategy

PUBMED

("Aorta/surgery"[MAJR] OR "Aortic Aneurysm, Thoracic/surgery"[MAJR] OR "Aortic Dissection/surgery"[Majr:NoExp] OR aortic surgery[tiab] OR aortic arch surgery[tiab] OR aortic replacement surgery[tiab] OR cardiac surgery[tiab] OR aortic dissection*[tiab] OR aortic operation*[tiab])

AND

("Hemostatics"[Mesh] OR "Blood Transfusion"[Mesh] OR "Antifibrinolytic Agents"[Mesh] OR "Blood Coagulation Factors"[Mesh] OR "Aprotinin"[Mesh] OR "Deamino Arginine Vasopressin"[Mesh] OR "Iron"[Mesh] OR "Erythropoietin"[Mesh] OR "Operative Blood Salvage"[Mesh] OR "cryoprecipitate coagulum" [Supplementary Concept] OR hemostatic*[tiab] OR haemostatic*[tiab] OR coagulation*[tiab] OR coagulopath*[tiab] OR blood management[tiab] OR prothrombin complex concentrate*[tiab] OR cryoprecipitate[tiab] OR Factor VIII[tiab] OR desmopressin[tiab] OR DDAVP[tiab] OR antifibrinolytic*[tiab] OR tranexamic acid[tiab] OR aprotinin[tiab] OR aminocaproic acid[tiab] OR EACA[tiab] OR cell salvage[tiab] OR cellsaver[tiab] OR autologous transfusion[tiab])

AND

("Randomized Controlled Trial" [Publication Type] OR "Cohort Studies"[Mesh] OR random*[tiab] OR prospective stud*[tiab] OR retrospective stud*[tiab] OR trial[tiab] OR comparison[tiab])

NOT

("Case Reports" [Publication Type] OR "Comment" [Publication Type] OR "Editorial" [Publication Type] OR "Letter" [Publication Type] OR letter[ti] OR comment*[ti] OR editorial[ti] OR case report[ti])

NOT

(("Animals"[MeSH Terms] OR "models, animal"[MeSH Terms] OR "Animal Experimentation"[MeSH Terms] OR "rodent"[Title] OR "rabbit"[Title] OR "mice"[Title] OR "mouse"[Title] OR "murine"[Title] OR "rat"[Title] OR "rats"[Title]) NOT "Humans"[MeSH Terms])

NOT

("Child"[Mesh] NOT "Adult"[Mesh]) NOT (pediatric*[ti] OR paediatric*[ti])

EMBASE (OVID):

Database(s): Embase Classic + Embase 1947 to July 01 2024
Search strategy:

#	Searches
1	exp *thoracic aortic surgery/ or aortic surgery/ or *thoracic aorta aneurysm/su or *aortic dissection/ or (aortic surgery or aortic arch surgery or aortic replacement surgery or cardiac surgery or aortic dissection* or aortic operation*).ti,ab,kf.
2	exp hemostatic agent/ or blood transfusion/ or blood autotransfusion/ or thrombocyte transfusion/ or fibrinogen concentrate/ or exp antifibrinolytic agent/ or aprotinin/ or desmopressin/ or iron/ or erythropoietin/ or blood salvage/ or cryoprecipitate/ or prothrombin complex/ or (hemostatic* or haemostatic* or coagulation* or coagulopath* or blood management or prothrombin complex concentrate* or cryoprecipitate or Factor VIII or desmopressin or DDAVP or antifibrinolytic* or tranexamic acid or aprotinin or aminocaproic acid or EACA or cell salvage or cellsaver or autologous transfusion).ti,ab,kf.
3	randomized controlled trial/ or cohort analysis/ or prospective study/ or retrospective study/ or major clinical study/ or (random* or prospective stud* or retrospective stud* or trial).ti,ab,kf.
4	1 and 2 and 3
5	limit 4 to conference abstract status
6	4 not 5
7	exp child/ not exp adult/
8	(pediatric* or paediatric*).ti.
9	case report/ or letter/ or editorial/ or note/ or (letter or comment* or editorial or case report).ti.
10	(exp animal/ or exp animal experiment/ or exp animal model/ or exp veterinary medicine/ or (animal* or monkey* or sheep or ovine or lamb or lambs or goat* or pig or pigs or swine or porcine or pup or pups or dog or dogs or canine or bitch* or beagle or feline or rodent* or rabbit* or rat or rats or mice or mouse or murine or cow or cows or horse or horses or ape or apes or gorilla or gorillas).ti,ab,kw.) not human/
11	6 not 7 not 8 not 9 not 10

Cochrane Central Register of Controlled Trials
Issue 6 of 12, June 2024

ID	Search
#1	aort* near/3 surgery
#2	(aortic dissection* or aortic operation*):ti,ab,kw
#3	(cardiac surgery):ti
#4	#1 or #2 or #3
#5	(hemostatic* or haemostatic* or coagulation* or coagulopath* or blood management or prothrombin complex concentrate* or cryoprecipitate or Factor VIII or desmopressin or DDAVP or antifibrinolytic* or tranexamic acid or aprotinin or aminocaproic acid or EACA or cell salvage or cellsaver or autologous transfusion):ti,ab,kw
#6	#4 and #5 in Trials
#7	(clinicaltrials or ICTRP or WHO portal or CINAHL):so
#8	#6 and #7
